# Case Report: Multimodality Imaging as a Lifeline for Fatal Localization of Valsalva Sinus Fibroelastoma

**DOI:** 10.3389/fcvm.2021.683534

**Published:** 2021-05-31

**Authors:** Snezana Tadic, Aleksandra Ilic, Maja Stefanovic, Anastazija Stojsic-Milosavljevic, Tanja Popov, Marija Bjelobrk, Aleksandra Milovancev, Nebojsa Maksimovic, Patrik Drid

**Affiliations:** ^1^Faculty of Medicine, University of Novi Sad, Novi Sad, Serbia; ^2^Department of Electrocardiography, Institute of Cardiovascular Diseases of Vojvodina, Sremska Kamenica, Serbia; ^3^Faculty of Sport and Physical Education, University of Novi Sad, Novi Sad, Serbia

**Keywords:** multimodality imaging, papillary fibroelastoma, Valsalva sinus, acute coronary syndrome, ostium, left coronary artery, cardiac surgery

## Abstract

**Background:** Papillary fibroelastomas are rare benign heart tumors, and is most likely to involve the cardiac valves. We will present an extremely rare localization of a large Valsalva sinus fibroelastoma, with occasional left coronary artery ostial obstruction presented as an acute coronary syndrome. The tumor was removed surgically and histologically confirmed as papillary fibroelastoma. This review points to the crucial importance of multidisciplinary team decision and multimodality imaging methods for diagnosing the fibroelastoma, determination of size, and localization, which avoided complications of fatal embolization during an invasive procedure.

**Case Summary:** A healthy 55-year-old male with vigorous physical daily training and exercise was admitted to the acute coronary syndrome emergency department. Shortly after admission, expert transthoracic echocardiography was performed. Computed tomography of the chest observed a large irregular hypodense tumor-like lesion in the bulbar aorta that was occasionally prolapsing into the left main coronary artery ostium and which corresponded to fibroelastoma. A few hours after admission, an emergency cardiac surgery was performed with the excision of a Valsalva sinus tumor (size 2 × 2 cm) located between the right and left coronary cusp of the aortic valve.

**Conclusions:** Focus cardiac ultrasound should be performed for any acute coronary syndrome because of the possible Valsalva sinus fibroelastoma etiology. Its localization next to the left main coronary artery ostium is rare, and dangerous. The timely diagnosis can be made by the multimodality imaging method, however, the final diagnosis will be made pathohistologically. Early cardiac surgery may be a necessitated recourse for these patients in order to prevent a fatal outcome.

## Introduction

Cardiac papillary fibrolestomas (CPF) are sporadic benign heart tumors, mostly asymptomatic, and its diagnosis has increased with the development of high-resolution imaging techniques (1, 2). In the literature, there are not many described cases of CPF with the presentation of acute coronary syndrome, and some ended as a fatal outcome (3–5). Our case of CPF had a good ending.

## Case Presentation

A healthy 55-year-old male with vigorous physical daily training and exercise was admitted to the acute coronary syndrome emergency department. The symptoms were sudden onset of upper back pain with propagation in the chest, dizziness, and generalized weakness, which appeared after intense physical activity (2 h of exercise in the gym).

Upon admission, the patient was conscious, oriented, afebrile, hypotensive. Diffuse ST depression and elevation of the AVR lead were detected by ECG and a sizeable mobile formation in the initial part of the ascending aorta by focus cardiac ultrasound. The inflammation parameters were in the reference range, while cardiac enzymes were mildly elevated.

**Figure 1:**
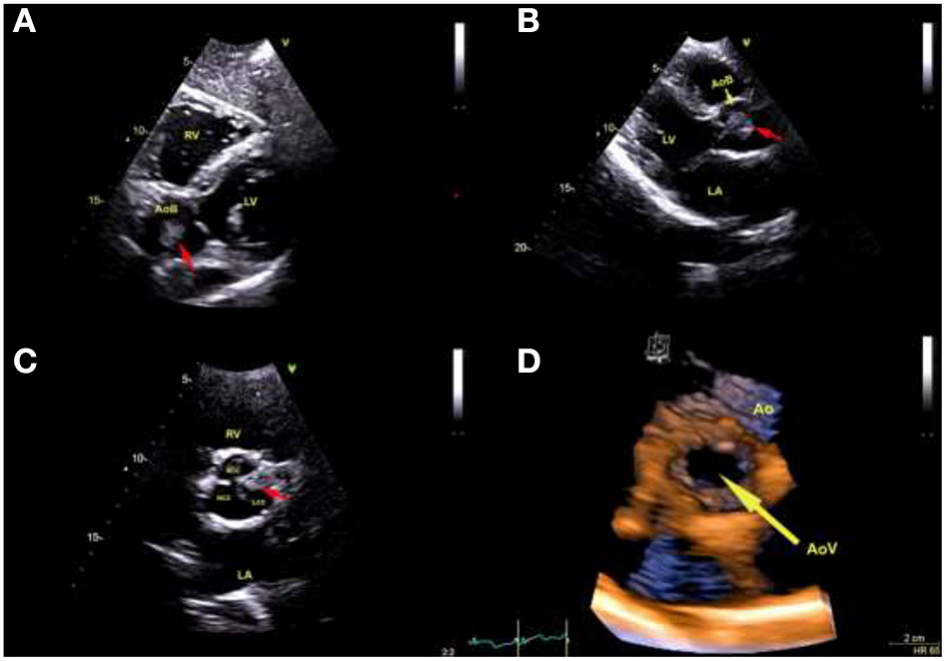
Transthoracic echocardiography: **(A)** Subxyphoid view shows pedunculated mass (red arrow) localization in Valsalva sinus. **(B)** Long-axis view measuring tumor size 1.9 × 1.5 cm (red arrow). **(C)** Short-axis view at aortic valve level demonstrates tumor (red arrow) originating immediately after the separation of the left and right coronary cusp. **(RV)** Right ventricle. **(LV)** Left ventricle. **(AoB)** Aortic bulb. **(LA)** Left atrium. **(RCC)** Right coronary cusp. **(NCC)** Noncoronary cusp. **(LCC)** Left coronary cusp. **(D)** Transthoracic three-dimensional echocardiography: view of the aortic valve (yellow arrow) from the aortic side, which shows the aortic cusps' normal appearance. **(AoV)** Aortic valve. **(Ao)** Aorta.

Shortly after admission, transthoracic echocardiography was performed. An irregular round mobile mass (dimensions 1.9 × 1.5 cm) was seen in the lumen of the aortic bulb, attached immediately after the separation of the left and right aortic coronary cusp, that fluttered in the aortic lumen and didn't enter the left ventricle (Figures 1A–C). There were no wall motion abnormalities and no other structural valve abnormalities noted including aortic stenosis or regurgitation (Figure 1D). Differentially diagnostic, the formation corresponded most closely to the tumor, especially fibroelastoma, with the rare localization in the Valsalva sinus.

**Figure 2:**
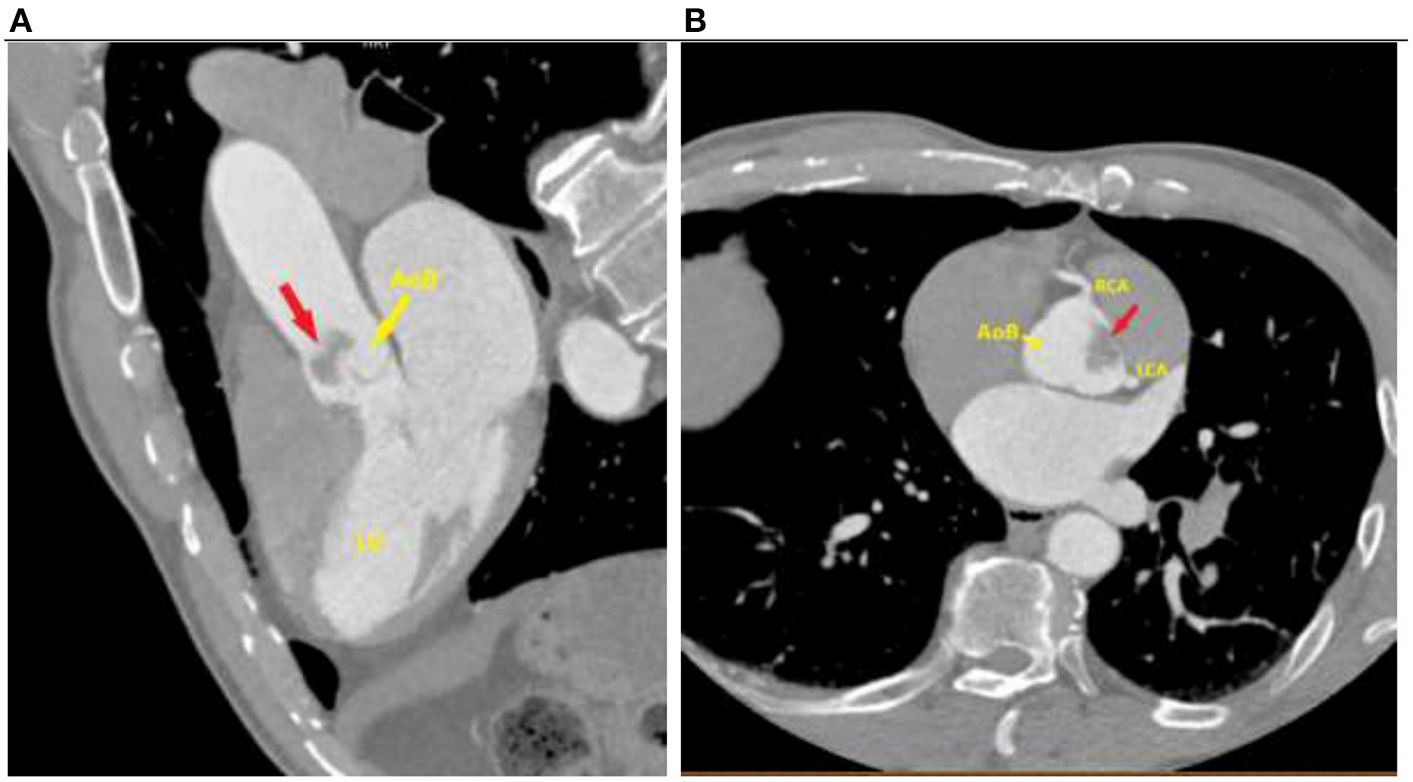
Computed tomography of the chest: **(A)** A large fibroelastoma (red arrow) in the aortic bulb. **(B)** Cross-section showing localization of fibroelastoma (red arrow) right next to the left coronary artery ostium. **(AoB)** Aortic bulb. **(LV)** Left ventricle. **(LCA)** Left coronary artery. **(RCA)** right coronary artery.

In order to have a more precise diagnosis, a chest CT scan was performed just after echocardiography. It observed a large irregular hypodense tumor-like lesion in the bulbar aorta that was occasionally prolapsing into the left main coronary artery ostium and which corresponded to fibroelastoma (Figure 2).

**Figure 3:**
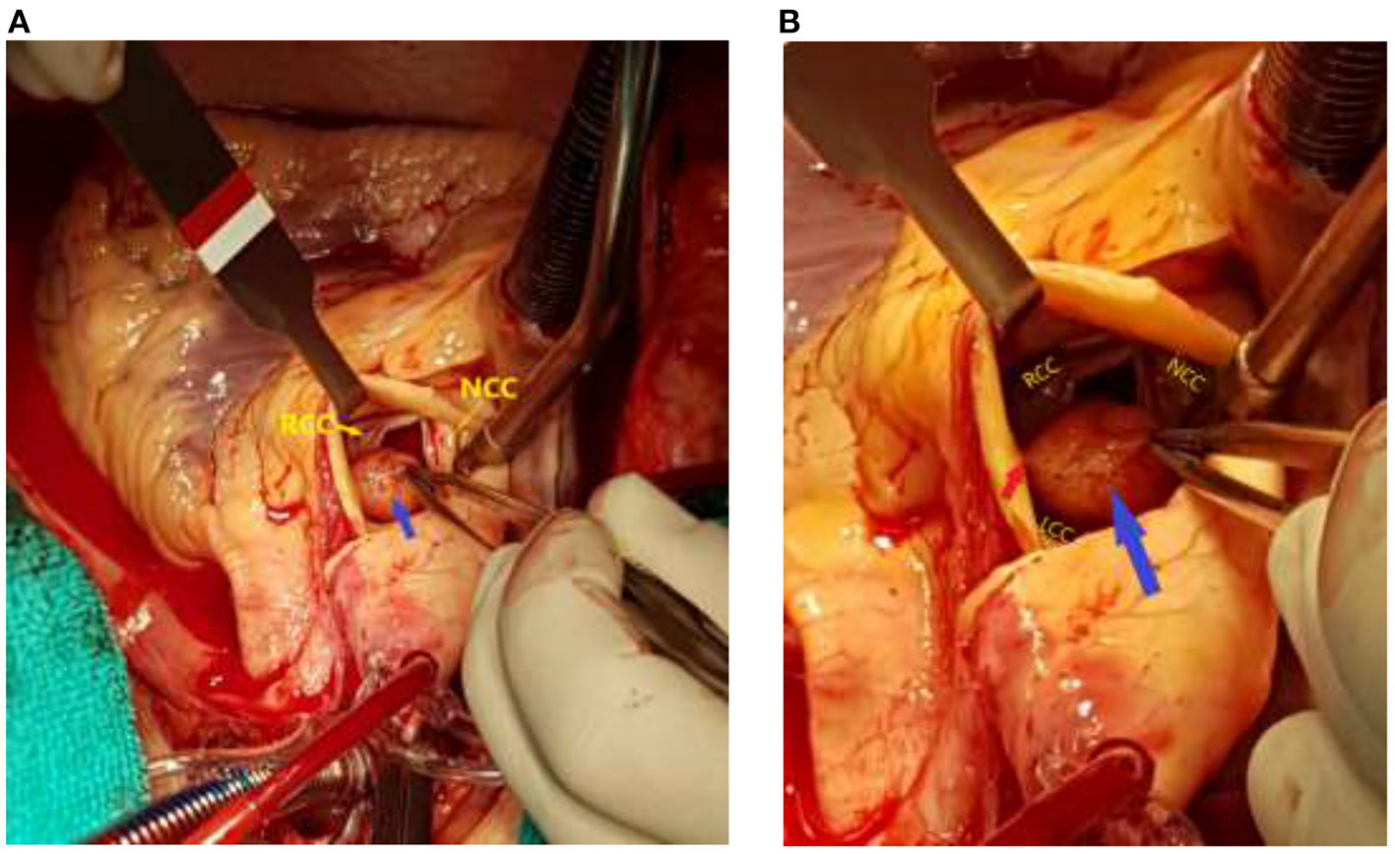
Surgical view after transverse aortotomy: **(A)** Fibroelastoma (blue arrow) localization in the Valsalva sinus. **(B)** A tumor (blue arrow) was attached by a thin stalk (red arrow) between the right and left coronary aortic cusps at the aortic side. Notice normal anatomical preservation of the aortic valve leaflets. The left coronary cusp was hidden behind the tumor. **(RCC)** Right coronary cusp. **(NCC)** Noncoronary cusp. **(LCC)** Left coronary cusp.

The multidisciplinary (“heart”) team decided not to preformed coronary angiography, as the tumor's localization was at high risk of systemic embolization. A few hours after admission, an emergency cardiac surgery was performed with the excision of a Valsalva sinus tumor (size 2 × 2 cm) located between the right and left coronary cusp of the aortic valve. In contrast, the aortic valve leaflets inspection determined their anatomical preservation (Figure 3).

**Figure 4:**
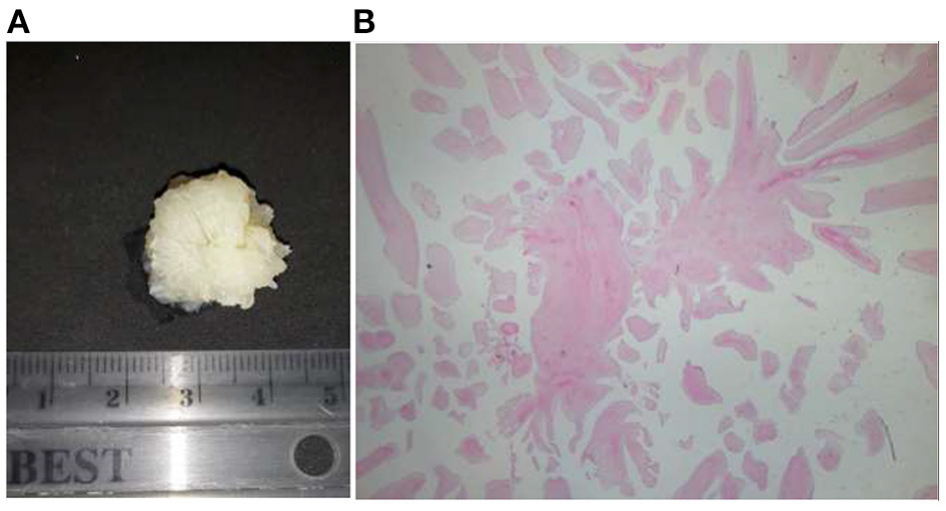
Pathological exam of papillary fibroelastoma: **(A)** Gross specimen. **(B)** Histological finding stained by hematoxylin-eosin method (a fragment from a tumor nodule constructed of numerous papillary formations lined with endothelial cells while a stromal papilla is constructed of a homogeneous, hypocellular portion of edematous connective tissue) on a low magnification.

Localization of the tumor was along the very left main coronary artery ostium. A pathohistological examination made the final diagnosis of papillary fibroelastoma (Figure 4).

The surgery and postoperative course were without complications. The patient had complete resolution of all ECG abnormalities and was discharged in a good general condition a week after the admission. After 1 year follow-up, control echocardiography revealed no more tumor mass. A detailed timeline from the onset of symptoms in the patient to his discharge is provided (Table 1).

**Table 1:** Timeline from symptoms onset to discharged.

**TIMELINE**	**Symptom onset to discharge**
22 February 2020 09:15 PM	Developed chest pain
22 February 2020 09:42 PM	Admission to the emergency department
22 February 2020 10:05 PM	Cardiac Focus Ultrasound
22 February 2020 10:20 PM	Admission to Coronary Care Unit
22 February 2020 10:40 PM	Transthoracic Echocardiography
22 February 2020 11:15 PM	Computed Tomography of the chest
22 February 2020 11:55 PM	Multidisciplinary (“heart”) team decision
23 February 2020 00:30 AM	Cardiac surgery
24 February 2020 09:35 AM	Histological diagnosis of papillary fibroelastoma
02 March 2020 01:30 PM	Discharged

The patient perspective was satisfactory, with prompt and complete recovery and no recurrence of similar symptoms. The quality of life has gotten better, especially since he returned to daily training in the gym after a few months of hospitalization. After 1 year follow-up, control echocardiography revealed no more tumors mass.

## Discussion

Papillary fibroelastomas are the third most common benign heart tumors, just behind myxomas and lipomas, covering a total of 10% of all benign primary cardiac tumors (3). It is also described as “tumor-like lesions” because histogenesis can be associated with organized microthrombi, continuous turbulent blood flow, and endothelial injury, as with neoplasm and congenital etiology too (2, 3, 6, 7). They are mostly asymptomatic and accidentally diagnosed due to high-resolution imaging methods. Although benign, a tumor can cause embolic complications (mostly cerebral), acute myocardial infarction, syncope, ventricular arrhythmias, and sudden cardiac death (1, 2).

CPF is the most common primary cardiac valvular tumor, and the non-valvular endocardial location is infrequent (7). In recent literature, only a few cases of CPF have been described in the Valsalva sinus, while just over twenty cases an acute coronary syndrome (3–5, 7). The majority of these patients had coronary embolization from the CPF surface, while less a dynamic obstruction of the coronary arteries ostia by the tumor itself (3–5, 7). Unfortunately, some of these cases were diagnosed by autopsy because the patients had been treated as sudden cardiac death (1). Some cases ended lethally during a coronary angiography due to iatrogenic embolization (7). This strongly confirms the importance of the focus cardiac ultrasound in acute coronary syndrome (8). Surgery is indicated to patients who have symptoms of the disease or developed complications and those where the localization is near the coronary arteries ostia, regardless of the symptoms (1, 2, 7).

Our opinion is that it was beneficial for the patient that CPF was diagnosed by the multimodality imaging method, which confirmed the size and the localization near the left coronary artery ostium. The obstruction of the left coronary artery ostium by the tumor prolapsing was the cause of the acute coronary syndrome. This is supported by the typical ECG abnormalities for left main stenosis, as well as slightly elevated cardiac enzymes. By avoiding urgent angiography in acute coronary syndrome, we have reduced possible and fatal embolization. The localization of the formation itself excluded thrombus or endocarditis (in the direction of blood flow, not the opposite), especially since the coagulation and inflammatory parameters were within the reference range, while cardiac enzymes were mildly elevated. Also, the patient has not had any symptoms or signs of embolic complications so far. Valsalva sinus is a rather rare localization for other primary heart tumors, because myxomas are most often localized on the interatrial septum and atria, and lipomas in the heart cavities (1, 6, 7). Differentially diagnostic, Lambl's excrescences develop at the valvular coaptation sites of the heart which are seen as thin, hypermobile, filiform strand and never growths to this size.

## Conclusion

This case report confirms the important role of cardiac focus ultrasound in all urgent cardiac conditions, as well as the multidisciplinary (“heart”) team decision. Multimodality imaging is a crucial for the diagnosis of the CPF, as well as its localization, size, and mechanism of development of acute coronary syndrome, by the tumor itself prolapsing into the left coronary artery ostium. Large cardiac tumors, especially localizations of Valsalva sinus, must be removed surgically, and the final diagnosis is made only histologically (1).

## Limitations

The images were taken bedside, on a portable echocardiographic device, because the patient was on continuous ECG monitoring in the Coronary Care Unit. Better information would be obtained using transesophageal echocardiography and/or MRI. The use of transesophageal echocardiography was limited by the epidemiological situation due to COVID-19, while MRI was not available to us at the time.

## Data Availability Statement

The raw data supporting the conclusions of this article will be made available by the authors, without undue reservation.

## Ethics Statement

The studies involving human participants were reviewed and approved by Institute of Cardiovascular diseases of Vojvodina. The patients/participants provided their written informed consent to participate in this study. Written informed consent was obtained from the individual(s) for the publication of any potentially identifiable images or data included in this article.

## Author Contributions

ST, AI, MS, AS-M, TP, and MB wrote the article. ST, AI, MS, AS-M, TP, MB, AM, NM, and PD designed the study, analyzed the data, discussed the results, and reviewed and approved the article. All authors contributed to the report and approved the submitted version.

## Conflict of Interest

The authors declare that the research was conducted in the absence of any commercial or financial relationships that could be construed as a potential conflict of interest.

## Supplementary Material

Supplementary Movie 1 Transthoracic echocardiography, a subxyphoid view (indicate the Figure 1A).

Supplementary Movie 2 Transthoracic echocardiography, a long-axis view (indicate the Figure 1B).

Supplementary Movie 3 Transthoracic echocardiography, a short-axis view (indicate the Figure 1C).

Supplementary Movie 4 Transthoracic three-dimensional echocardiography view of the aortic valve (indicate the Figure 1D).
